# Timing Matters? Impact of Early Sperm Processing on Motile Sperm Recovery, Fertilization, Blastocyst Rate, and Pregnancy Outcomes in IUI and IVF †

**DOI:** 10.3390/jcm14228094

**Published:** 2025-11-15

**Authors:** Kiana Loo, Isabelle Mason, Rebecca Chung, Samantha Sechler, Kathryn Coyne, Joseph Findley, Rachel Weinerman, Rebecca Flyckt, Sung Tae Kim

**Affiliations:** 1 Division of Reproductive Endocrinology and Infertility, Case Western Reserve University School of Medicine, Cleveland, OH 44106, USA; ksl64@case.edu (K.L.); isabelle.mason@uhhospitals.org (I.M.); kathryn.coyne@uhhospitals.org (K.C.); joseph.findley@uhhospitals.org (J.F.); rachel.weinerman@uhhospitals.org (R.W.); rebecca.flyckt2@uhhospitals.org (R.F.); 2 Department of Reproductive Endocrinology and Infertility, University Hospitals, Beachwood, OH 44112, USA; 3 Department of Reproductive Endocrinology and Infertility, University of Washington School of Medicine, Seattle, WA 98195, USA

**Keywords:** sperm preparation, male factor infertility, assisted liquefaction, asthenozoospermia

## Abstract

**Background/Objectives:** Efficient sperm separation is critical for Assisted Reproductive Technology (ART). This study evaluates how assisted liquefaction (AL), a faster sperm preparation technique, affects motile sperm recovery and ART outcomes. **Methods:** A prospective pair analysis to address the efficiency of AL and retrospective cohort analysis of IUI and IVF/ICSI cycles were performed. **Results:** The average time to perform AL was 3 min, in comparison to the 15 to 30 min for traditional liquefaction (TL) until initiating sperm preparation. The total motility (*p* = 0.252) and progressive motility (*p* = 0.227) of the sperm samples were about 4% higher in the AL group although there was no significant difference. No significant differences were observed between AL and TL in fertilization (80.0% vs. 81.9%, *p* = 0.297), blastocyst rates (48.2% vs. 48.1%, *p* = 0.595), positive pregnancy (70.5% vs. 75.0%, *p* = 0.667), clinical pregnancy (65.9% vs. 68.4%, *p* = 0.841), or live birth (52.3% vs. 51.3%, *p* = 0.831) outcomes in IVF. The Positive pregnancy (*p* = 0.669) and clinical pregnancy (*p* = 0.841) of IUI were not significantly different between both groups. **Conclusions:** AL offers similar results to TL while reducing preparation time without interrupting ART outcomes. AL may have benefit to asthenozoospermia cases for ART due to reduced exposure time of sperm to semen that may prevent sperm quality impact.

## 1. Introduction

Male factor infertility is estimated to account for 40–50% of all infertility cases worldwide and affects 12% of men in the United States between 15 and 44 years old [1,2,3,4]. Semen preparation and analysis provides insight into potential etiologies of male factor infertility and optimizes semen samples for intrauterine insemination (IUI), intracytoplasmic sperm injection (ICSI), and in vitro fertilization (IVF). One of the most important steps in the preparation and analysis is the expeditious separation of motile sperm from its seminal plasma. The traditional method of sperm separation, as outlined in the 2021 World Health Organization (WHO) manual [5], involves incubating the semen sample at 37 °C for 15 to 30 min. Failure to adhere to this time limit can severely impact motility, morphology, and other qualities of the sperm sample [6,7,8]. Furthermore, deficiencies in these qualities may impair fertilization and subsequent embryo development [2,8,9].

Existing sperm preparation methods include simple washing, direct swim-up, discontinuous density gradients, wash and centrifugation, and magnetic activating cell sorting (MACS) [8,9,10,11,12,13]. These all occur after proper liquefaction is achieved and allow for isolation of the most motile and viable sperm by manipulating mechanical, biochemical, or physiological separation mechanisms [12]. However, these methods are highly dependent on initial sperm quality and increase the time and cost of the sperm preparation process [4]. For samples with asthenozoospermia, or reduced sperm motility, multiple rounds of these methods are often attempted to achieve the same amount of quality sperm as a normospermic counterpart. These multiple rounds of processing increase the risk of mechanical shearing and excessive reactive oxidative species generation, potentially compromising already suboptimal sperm samples [10].

Even in normal semen samples, it has been shown that an increase in manipulation of semen samples can cause an increase in oxidative stress (OS), that can negatively affect fertilization and embryo development due to DNA damage [14]. Thus, a recent area of andrology and ART research has focused on newer techniques, such as microfluidic sorting, that yield decreased DNA fragmentation and allows for the self-selection of highest quality sperm [15,16,17].

There are multiple other aspects of sperm preparation, however, that leave sperm vulnerable including: exposure to light, prolonged time in culture media, pH and temperature. While many studies are evaluating the effects of different methods of sperm selection (e.g., density, swim up, and various microfluidic techniques as mentioned above) to decrease DNA fragmentation, there has been less emphasis on the other potential contributors such as prolonged time in culture media and temperature [18]. Balasuriya et al. assessed the effects of short-term storage at 37 °C and found that even within one hour, sperm that had been exposed to heat showed increased oxidative stress compared to room-temperature counterparts [19].

It is from this data that our lab proposed a novel method of sperm preparation to decrease the amount of time sperm is exposed to environmental factors that may increase oxidative stress and decrease sperm quality: assisted liquefaction.

Assisted liquefaction (AL), is a new technique by which pre-warmed sperm wash media is immediately added to semen samples before processing, offering a quicker alternative to the initial step of sperm preparation for IUI and IVF. AL warms the sperm wash to the same temperature at which semen samples would otherwise be incubated, but it reduces the initial preparation time from 30 min down to 3 min. This study seeks to evaluate the effect of the AL technique in sperm preparation for IVF and IUI, specifically on motile sperm recovery, fertilization, blastocyst development, and pregnancy outcomes.

## 2. Materials and Methods

### 2.1. Pilot Study

**Figure 1:**
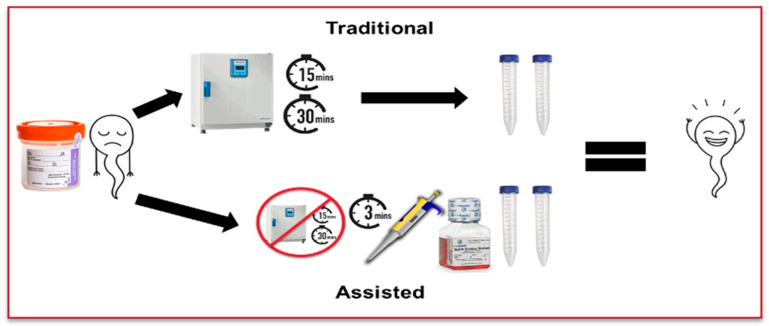
Separation of samples into traditional and assisted liquefaction techniques.

Twenty fresh semen samples were randomly selected from patients in a single academic fertility center in Northeast Ohio. Inclusion criteria included at least 2.5 mL of volume and 10 million motile sperm, and only azoospermic samples were excluded. Each sample was divided in half right after receiving the sample; one half was prepared via AL while the other half was prepared via TL. As outlined by the WHO 6th edition, the TL technique liquefies each sample for 15–30 min at 37 °C [5]. The AL technique involves adding pre-warmed sperm wash media at 37 °C (Multipurpose Handling Medium, Fujifilm Irvine Scientific, Santa Ana, CA, USA) at twice the semen volume to the sample before sample processing. After liquefaction, the same sperm preparation process was performed in both groups: each sample was overlaid onto 2 mL of 90% density gradient (Fujifilm Irvine Scientific, CA, USA) with no more than 1.5 mL of sample per tube. The sample was centrifuged at 400× *g* for 15 min. The supernatant was then removed, and samples from multiple tubes were combined if needed. After additional washing, a 5 μL aliquot from both groups was counted using a pre-warmed MicroCell as a post-wash (Figure 1). The total motile sperm, total motility, and progressive motility were assessed in both groups by paired *t*-test. A *p* value of <0.05 was considered significant. Graph Pad Prism (version 10) was used for statistical analysis.

### 2.2. Retrospective Study

The second component of this study was a retrospective cohort study that examined both IUI and IVF cycles using fresh sperm at the same single academic-based fertility center between May 2021 and April 2022 that used AL or TL preparation techniques. Inclusion and exclusion criteria were the same as above. The two cohorts were defined based on the type of sperm preparation performed: AL or TL. Data was collected through a retrospective chart review. Both AL and TL techniques are the same as performed in the pilot study. Additional processing steps that follow the initial separation protocols remain the same for both methods. Following the completion of sperm preparation, IUI was initiated immediately after the completion of sperm preparation, while ICSI as the only method of fertilization is initiated 3–5 h after oocyte retrieval as previously described [20].

The primary outcomes in this study include the prevalence of positive pregnancy and clinical pregnancy following either fresh embryo transfers or IUI. Clinical pregnancy is defined as the presence of intrauterine gestation on ultrasound, whereas total pregnancy is defined as any patient with a positive human chorionic gonadotropin (hCG). Additional outcomes analyzed include normal fertilization, abnormal fertilization, and blastocyst rates. Fertilization status was confirmed 16–20 h after fertilization with normal fertilization confirmed by the presence of two pronuclei at this time point and abnormal fertilization defined by either one or three or more pronuclei. Blastocyst rate and stage were determined using the Gardner embryo grading system and defined as the total number of usable blastocysts per total number of normally fertilized embryos. *T*-tests and Fisher Exact tests were used to analyze differences between outcomes from the AL and TL techniques. A *p* value of <0.05 was considered significant.

## 3. Results

### 3.1. Pilot Study: Assessment of Motile Sperm Recovery

The average time to perform AL was 3 min, in comparison to the 15 to 30 min for TL. The average difference in total motility of the sperm samples before and after TL was 0%, whereas the change in total motility after AL applied was a 4% increase in motility, which did not prove to be statistically significant (*p* = 0.252). Similarly, the average change in progressive motility of the sperm in samples before and after TL was 0% in comparison to the 4% increase in progressive motility in sperm samples prepared via AL, but no statistical difference (*p* = 0.227). Finally, the average change in number of total motile sperm in samples subjected to TL was 0, whereas the number of total motile sperm in samples prepared via AL was 1.75 × 10^6^ sperm (*p* = 0.252) although there was no significant difference (Table 1). Additionally, samples processed via TL were observed to contain more non-motile sperm after processing than the assisted liquefaction technique.

### 3.2. Retrospective Study: Comparison of Fertilization and Blastocyst Rates in IVF Cycles

To address the effect of AL technique-driven sperm preparation on IVF outcome, we analyzed 479 IVF cycles. The average age of female, BMI (body mass index), or AMH (anti-mullerian hormone) was not significantly different in both groups (Table 2).

The normal fertilization rates in the AL cohort (n = 216) and the TL cohort (n = 263) were 80.0% and 81.9% (*p* = 0.297), abnormal fertilization rates between the AL and TL cohorts were 1.6% and 1.3% (*p* = 0.484), respectively. Finally, good quality of blastocyst rates between the AL and TL cohorts were 48.2% and 48.1% (*p* = 0.595) with a *p*-value that was not statistically significant (Table 3). The data suggested that there is no impact of AL technique-driven sperm preparation on fertilization or blastocyst rate in IVF cycles.

### 3.3. Retrospective Study: Comparison of Pregnancy Outcomes in IVF and IUI Cycles

Through 120 fresh embryo transfer (ET) cycles, we also assessed IVF pregnancy outcomes. Average age of female or endometrial thickness at the time of trigger was not significantly different in both groups. The pregnancy outcomes, positive and clinical pregnancy outcomes for ET cycles prepared via the AL technique were not statistically different from the TL technique (70.5% vs. 75.0%, *p* = 0.667 and 65.9% vs. 68.4%, *p* = 0.841). The live birth rate via the AL technique was also not statistically different from the TL technique (52.3% vs. 51.3%, *p* = 0.831) (Table 4).

A total of 1072 IUI cycles were analyzed that had at least 5 × 10^6^ motile sperm. Average age of female was not significantly different in both groups (*p* = 0.886). Similarly to IVF outcome, the positive pregnancy rates between the AL and TL cohorts were 14.5% and 13.6%, respectively, which was a statistically insignificant difference (*p* = 0.669). The clinical pregnancy rate for IUI cycles that were prepared via the AL technique was 11.9% in comparison to the 12.9% of the TL cohort, with a *p*-value (*p* = 0.841) that was not statistically significant (Table 5).

## 4. Discussion

Assisted liquefaction was found to not significantly impact total or progressive motility when compared to the traditional incubation technique. Similarly, there were no statistically significant differences in fertilization, blastocyst rates, or pregnancy outcomes between the assisted liquefaction technique and the traditional sperm technique. These results further support our previous findings that assisted liquefaction does not appear to impact the motility of prepared sperm nor its subsequent use in ART in a significant manner, suggesting assisted liquefaction may be a method of preparation that could be comparable to traditional incubation in the sperm preparation process.

Few studies comment on the impact of different sperm preparation techniques on clinical pregnancy and live birth outcomes [9,10,11,13,21]. Our data, however, looked at pregnancy outcomes from our sperm preparation method and found no difference in our small sample size compared to the traditional liquefaction technique, giving credence to its safety and potential validity. The novelty of assisted liquefaction however also means that while early findings are promising, more studies are needed to verify its impact on ART, pregnancy, and live birth outcomes.

There were several limitations to this study. The pilot study was limited by sample size and being performed at a single fertility center, necessitating repeated studies to reach sufficient power to determine statistically significant results. The retrospective cohort study was also limited by the inability to control for inherent biases in retrospective studies, including potential selection and information bias. This study did not stratify cohorts by male factor infertility or sperm sample motility. Additionally, other aspects of spermatozoa vitality associated with fertility outcomes were not evaluated, including reactive oxygen species and DNA fragmentation [8,9,21,22].

Nonetheless, these findings are important for the field of reproductive endocrinology and infertility because they can potentially optimize laboratory techniques without compromising the quality of care, thereby increasing the productivity needed to meet the growing demands of ART. Adequate liquefaction is important for the subsequent use of preparation methods like direct swim-up or density gradients. Given the growing discussion and research surrounding oxidative stress and DNA damage accrued during sperm preparation, improving the liquefaction process through assisted liquefaction may help to reduce sperm exposure to reactive oxidative species associated with repeated sperm preparation and prolonged exposure to seminal plasma [2,8]. A subgroup of patients that may greatly benefit would be those with asthenozoospermia, where AL would reduce exposure time in media and allow for less attrition through the preparation process [4,6]. Our team recently presented that sperm DNA fragmentation increases and sperm motility decreases over time in seminal plasma from collection to analysis [23]. This also suggests isolating motile sperm as soon as possible in semen to keep sperm integrity.

Future studies should include the impact of assisted liquefaction on sperm quality when used in conjunction with various sperm preparation methods, as well as randomized controlled trials that assess live birth rates and stratify cohorts by cause of infertility, indication for assisted reproductive technology, type or protocol of ART utilized, and semen analysis results, while assessing DNA fragmentation and oxidative stress. Specific research examining AL in an asthenozoospermic population is also needed.

## 5. Conclusions

Assisted liquefaction (AL) is a novel semen preparation technique that showed shortened preparation time compared to existing liquefaction techniques without impacting motile sperm recovery, fertilization, blastocyst and pregnancy rates among couples undergoing assisted reproductive technology. While this study’s sample was limited, AL may have benefits by reducing the motile sperm exposure time in semen and may efficiently reduce preparation duration as well as semen cryopreservation. Future research should continue to expand on the utility of AL across different types of male factor infertility and protocols of ART. Large, multicenter randomized control trials are needed to further study this preparation method and its role in the future of ART.

## Figures and Tables

**Table 1:** Differences in motility between assisted liquefaction techniques compared to traditional liquefaction.

Assisted Liquefaction (n = 20)	Average of Difference	Range of Difference	*p* Value
Total Motility	4%	−1 to 32%	*p* = 0.252
Progressive Motility	4%	−1 to 34%	*p* = 0.227
Total Motile Sperm	1.75 × 10^6^	−1.2 × 10^6^ to 13.5 × 10^6^	*p* = 0.252

**Table 2:** Patient’s demographic information in IVF cycle between TL and AL cohorts.

	AL	TL	*p* Value
# of IVF cycles	216	263	
Average age of female (years)	35.6	35.2	*p* = 0.384
Average * BMI of female (kg/m^2^)	27.2	26.5	*p* = 0.226
Average ** AMH of female (ng/mL)	3.75	3.03	*p* = 0.055

* BMI, body mass index; ** AMH, anti-mullerian hormone.

**Table 3:** Fertilization and blastocyst rate for IVF-ICSI between traditional and assisted liquefaction cohorts.

	AL	TL	*p* Value
# of IVF cycles	216	263	
Average number of oocytes	13.9	12.7	*p* = 0.079
Fertilization Rate (%)	80.0	81.9	*p* = 0.297
Abnormal Fertilization Rate (%)	1.6	1.3	*p* = 0.484
Blastocyst Rate (%)	48.2	48.1	*p* = 0.595

**Table 4:** Clinical outcomes of IVF between traditional and assisted liquefaction cohorts.

	AL	TL	*p* Value
# of Fresh ET	44	76	
Average age of female (years)	34.6	35.5	*p* = 0.287
Average endometrial thickness (mm)	9.5	10.2	*p* = 0.091
Positive Pregnancy Rate (%)	70.5	75.0	*p* = 0.667
Clinical Pregnancy Rate (%)	65.9	68.4	*p* = 0.841
Live Birth Rate (%)	52.3	51.3	*p* = 0.831

**Table 5:** Clinical outcomes of IUI between traditional and assisted liquefaction cohorts.

	AL	TL	*p* Value
# of IUI cycles	512	560	
Average age of female (years)	34.7	34.8	*p* = 0.886
Positive Pregnancy Rate (%)	14.5	13.6	*p* = 0.669
Clinical Pregnancy Rate (%)	11.9	12.9	*p* = 0.841

## Data Availability

The raw data supporting the conclusions of this article will be made available by the authors on request.
